# In Young Adults with COVID-19, Obesity Is Associated with Adverse Outcomes

**DOI:** 10.5811/westjem.2020.5.47972

**Published:** 2020-06-15

**Authors:** Eric Steinberg, Ellsworth Wright, Beth Kushner

**Affiliations:** St. Joseph’s Health, Department of Emergency Medicine, Paterson, New Jersey

## Abstract

**Introduction:**

For patients with COVID-19, several characteristics have been identified that may be associated with adverse outcomes. However, there is a paucity of data regarding the effect of obesity on young adult patients with COVID-19. We sought to identify whether adverse outcomes are associated with obesity, particularly in COVID-19 patients 45 years and younger.

**Methods:**

This was a two-center, retrospective cohort study that included 210 patients. Eligible patients were between the ages of 18–45 years old, had tested positive for SARS-CoV-2 on real-time reverse transcription polymerase chain reaction via nasopharyngeal swab, and were not pregnant. Primary outcomes were defined as follows: 1) in-hospital mortality during the study period; 2) need for mechanical ventilation; and 3) admission to the hospital. We analyzed baseline characteristics of the cohort using descriptive statistics. Odds ratios (OR) were calculated to assess associations between outcomes and obesity, defined as body mass index (BMI) >30.

**Results:**

Of those patients who tested positive, 18 died during hospitalization (9%), 36 (17%) required mechanical ventilation, and 94 (45%) were admitted. Each of the primary outcomes was significantly associated with a BMI >30 (mortality OR = 6.29, 95% confidence interval [CI], 1.76–22.46, p = 0.0046; mechanical ventilation OR = 6.01, 95% CI, 2.5–14.48, p = 0.0001; admission OR 2.61, 95% CI, 1.49–4.58, p =.0008).

**Conclusion:**

Obesity appears to be an independent risk factor for poor outcomes in young patients with COVID-19. Future studies examining the clinical characteristics and risk factors of COVID-19 patients across large, diverse populations will strengthen our understanding of this novel and complex disease.

## BACKGROUND

In December 2019, a novel coronavirus was identified that has since changed the world as we know it. SARS-CoV-2 began infecting people in the Hubei Province in China and has since affected people on almost every continent. Experts identified the clinical syndrome caused by this virus as COVID-19, which primarily manifests as a respiratory illness that has high transmissibility, pathogenicity, morbidity, and mortality. Clusters of outbreaks continue to appear, most recently affecting the United States. Studies have identified comorbidities that may be associated with worse outcomes, including diabetes, hypertension, cardiovascular disease, chronic obstructive pulmonary disease, malignancy, and chronic liver disease.^1^–^2^ Clinicians in the US are taking notice of another trend – younger patients who are obese (defined as body mass index [BMI] equal to or greater than 30) appear to be at greater risk for adverse outcomes when contracting the virus. Two recently published articles identified obesity as a risk factor for COVID-19.^3^–^4^ This may not have been broadcasted as a known risk factor from the Chinese, South Korean, and Italian cohorts, as their obesity rates are significantly lower than US rates: 6.2%, 4.7%, 19.9%, respectively, compared to 36.2% in the US.^5^

The devastation that SARS-CoV-2 is causing is simply unprecedented in our lifetime. There is building evidence that identifies the clinical characteristics and features of this disease.^1^–^4^ High case-fatality rates have been reported in both China and Italy: 2.3% and 7.2%, respectively.^6^–^7^ More data is being reported on a daily basis that changes our approach to this growing pandemic. This highlights the need to identify any and all potential risk factors and/or clinical outcomes that may alter future clinical practice.

Obesity has been previously identified as a risk factor for disease severity in viral illnesses. During the H1N1 outbreak in 2009, numerous investigations identified a greater number of subjects with obesity admitted for in-patient care, those requiring mechanical ventilation, and overall mortality.^8^ This disproportionate effect of viral illnesses on obese patients identifies a potential risk factor that needs to be further investigated given the COVID-19 outbreak. We sought to identify whether adverse outcomes such as mortality, need for mechanical ventilation, or hospitalization are associated with obesity, particularly in COVID-19 patients 45 years and younger.

## METHODS

This retrospective cohort study was conducted at two sites: a high-volume, urban, academic, tertiary-care medical center and an affiliated suburban community hospital. Each chart was reviewed by at least two investigators. Sample charts were reviewed by all three principal investigators to assess interrater agreement. Although none of the three investigators were blinded to the study hypothesis, the chart elements of interest were clearly defined and objective, mitigating the need for interpretation of ambiguous elements. Patient information was de-identified to secure patient confidentiality. The study was reviewed and approved by a single institutional review board that reviews research for the health system.

Eligible patients were between the age of 18 and 45 years old who had presented to one of two emergency departments between March 8–April 4, 2020, and tested positive for SARS-CoV-2 on real-time reverse transcription polymerase chain reaction via nasopharyngeal swab. Patients who were pregnant (upon history or laboratory investigation) were excluded from the study. Although the decision to test a patient for SARS-CoV-2 was at each physician’s discretion on a case-by-case basis, they were expected to follow institutional policy in accordance with the Centers for Disease Control and Prevention’s “Priorities for Testing Patients with Suspected COVID-19 Infection.”^9^ Patients who were discharged from the hospital before test results returned were followed up via phone call by an emergency provider. Patients who were discharged and had not returned to the hospital system after the follow-up period were assumed to be alive and not admitted elsewhere.

A total of 210 patient charts were included in the study. Demographic data (age, gender, BMI) and the presence or absence of three primary outcomes (in-hospital mortality, need for invasive mechanical ventilation, and admission to hospital) were recorded. We analyzed baseline characteristics of the cohort using descriptive statistics. Odds ratios were calculated to assess associations between outcomes and BMI.

## RESULTS

Of 210 eligible patients, 18 died during hospitalization (9%), 35 (17%) required mechanical ventilation, and 94 (45%) were admitted to the hospital. Of 116 discharged patients, 103 (89%) were successfully followed up and confirmed to be alive in home-quarantine within one week of ED presentation. Of this group, one patient was reported to be admitted to another hospital.

Descriptive statistics by outcome are shown in the Table. Patients who died had a mean BMI of 37.97 (+/− 7.27) compared to 29.75 (+/− 6.21) for those who were alive at the end of the study period. Patients who required mechanical ventilation had a mean BMI of 35.72 (+/− 6.98) compared to 29.82 (+/− 6.95) for those who did not. Patients who were admitted to the hospital had a mean BMI of 32.47 (+/− 7.48) compared to 29.3 (+/− 6.49) for those who were discharged.

Each of the three primary outcomes were significantly associated with a BMI >30 as shown in the Figure (mortality OR = 6.29, 95% CI, 1.76–22.46, p = 0.0046; mechanical ventilation OR = 6.01, 95% CI, 2.5–14.48, p = 0.0001; admission OR 2.61, 95% CI, 1.49–4.58, p =.0008).

At the end of our study period, two patients who required mechanical ventilation (both with BMI >30) remain admitted to an intensive care unit. One was transferred to another hospital for extracorporeal membrane oxygenation. Therefore, that patient’s final disposition (i.e., discharge or death) is not known.

## DISCUSSION

Although previous studies representing different cohorts address obesity when describing clinical characteristics of COVID-19 patients, we are the first to address obesity as a potential independent risk factor for adverse outcomes specific to adults 45 years old and under with COVID-19. A recent, single-center study from New York University concluded that obesity in adults under 60 is a risk factor for hospital admission and need for intensive care but did not investigate mortality or need for mechanical ventilation.^10^ Until recently, obesity may have been overlooked as a meaningful risk factor, as countries such as China, South Korea, and Italy have far lower rates of obesity than the US. Our findings agree with evidence from the H1N1 outbreak in 2009, in which the presence of obesity was associated with poor outcomes including mechanical ventilation requirement and mortality.

## LIMITATIONS

As a two-center, retrospective case-control study, our study has limitations. The patient sample from this relatively small geographic area may not be representative of all other populations, and therefore may limit generalizability. In addition, co-morbid conditions such as diabetes or hypertension were not accounted for, which may have an impact on the clinical course of COVID-19. It is worth noting, however, that a substantial number of (43%) these young patients did not have a primary care physician and therefore were likely unaware of the presence of any co-morbid conditions. Of note, such comorbidities are often encompassed under the umbrella term “metabolic syndrome,” which many obese patients possess. Next, although we had a relatively high follow-up rate, it was assumed that discharged patients who did not return to the hospital system were alive and not admitted elsewhere, potentially impacting the accuracy of our results. Finally, this cohort likely represented a sicker group than the general population, as only patients who were selected for testing and tested positive were included in the study.

## CONCLUSION

Obesity appears to be an independent risk factor for poor outcomes in young patients with COVID-19. Future studies examining the clinical characteristics and risk factors of COVID-19 patients across large, diverse populations will strengthen our understanding of this novel and complex disease.

## Figures and Tables

**Figure:**
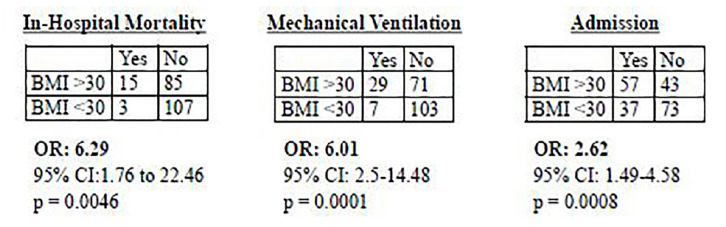
Odds ratio for BMI and primary outcomes. *BMI*, body mass index; *OR*, odds ratio; *CI*, confidence interval.

**Table:** Descriptive statistics by outcome.

Group	Mean BMI	SD	Sample size	Range
In-hospital mortality	37.97	7.27	18	24.98–58.48
Alive at end of study period	29.74	6.21	192	19.28–55.32
Required mechanical ventilation	35.72	6.98	36	23.94–58.48
Did not require mechanical ventilation	29.82	6.95	174	19.38–47.48
Admitted to hospital	32.47	7.28	94	20.76–58.48
Discharged from ED	29.3	6.49	116	19.38–47.48

*BMI*, body mass index; *SD*, standard deviation; *ED*, emergency department.
